# Colorectal perforation following anorectal manometry in a patient with anal stenosis post rectal prolapse repair: a rare complication

**DOI:** 10.1093/jscr/rjaf851

**Published:** 2025-12-04

**Authors:** Leanne Iorio, Monique Couto Matos, Benjamin Linkous, Marco Ferrara, Antonio Caycedo

**Affiliations:** Orlando Health Colon and Rectal Institute, 110 W Underwood St # A, Orlando, FL 32806, United States; Colon and Rectal Surgery, Orlando Health Orlando Regional Medical Center, 110 W Underwood St, Orlando, FL 32806, United States; Florida State University College of Medicine, 1115 West Call Street, Tallahassee, FL 32306, United States; Orlando Health Colon and Rectal Institute, 110 W Underwood St # A, Orlando, FL 32806, United States; Orlando Health Colon and Rectal Institute, 110 W Underwood St # A, Orlando, FL 32806, United States; General Surgery, University of Central Florida, 3400 Quadrangle Blvd, Orlando, FL 32817, United States; Colon and Rectal Surgery, Queen’s University, Etherington Building Room 202, 165 Barrie Street, K7L 2Z4, Kingston, ON, Canada

**Keywords:** anorectal manometry, rectal perforation, anal stenosis, nonoperative management, anticoagulation

## Abstract

Anorectal manometry (ARM) is a valuable diagnostic modality for evaluating anorectal disorders. While rare, complications can occur including rectal perforation. We report a case of iatrogenic rectal perforation following ARM in a 77-year-old female with multiple prior rectal prolapse surgeries who was receiving chronic anticoagulation therapy. The patient developed rectal bleeding immediately post-procedure with subsequent imaging demonstrating retroperitoneal and mediastinal air. Flexible sigmoidoscopy revealed a 30% circumference rectal wall defect. Despite the extensive injury, the patient was hemodynamically stable without peritonitis. Conservative management consisting of bowel rest, broad-spectrum antibiotics resulted in complete resolution. Serial endoscopic evaluation confirmed full mucosal healing. This case demonstrates the importance of individualized procedural risk assessment in patients with prior anorectal surgery and underscores the feasibility of conservative management in stable cases of ARM-related rectal perforation.

## Introduction

Anorectal manometry (ARM) assesses the functionality of the rectum and anal sphincters by measuring pressures, reflexes, and sensation. It is essential in evaluating conditions like fecal incontinence, chronic constipation, and anorectal pain syndromes [1]. While ARM is generally considered safe and minimally invasive, rare but serious complications, including rectal perforation, have been reported [2–4].

We present a rare case of rectal perforation following ARM in a patient with anal stenosis secondary to multiple rectal prolapse surgeries. Recurrent rectal prolapse is not uncommon. Although perineal procedures can be safely repeated, resection-based procedures carry the risk of leaving an ischemic segment between two anastomoses, especially when previous anastomosis are not resected [5]. This case highlights two key points: the need for tailored surgical planning in patients with recurrent prolapse and the importance for careful patient selection and technique during ARM to minimize the risk of iatrogenic injury.

## Case report

A 77-year-old female with a history of atrial fibrillation (on dabigatran), COPD, pulmonary fibrosis, coronary artery disease, multiple transient ischemic attacks, and rectal prolapse presented to the emergency department with hematochezia and fever one day after undergoing ARM.

Her surgical history included a resection rectopexy in 2006 and perineal proctectomy with sphincteroplasty in 2019 for recurrent rectal prolapse. Postoperatively, she developed anal stricture and fecal incontinence. ARM was performed as part of a diagnostic work up by her gastroenterologist; anticoagulation was not held prior to the procedure.

**Figure 1:**
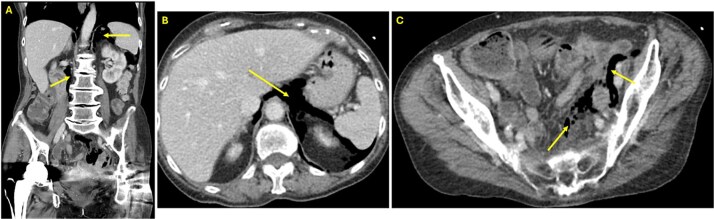
(A) Coronal view CT of the abdomen and pelvis showing free air in the retroperitoneum (arrows). (B) Axial view CT of the abdomen and pelvis at the level of the liver demonstrating free air in the retroperitoneum (arrow). (C) Axial view CT in the pelvis revealing free air in the colonic mesentery (arrows), consistent with extraluminal gas from rectal perforation.

At presentation to the Emergency Department, she reported painless rectal bleeding. On physical exam, her abdomen was nontender, with mild distension and minimal bleeding was noted at the anus. Laboratory findings demonstrated leukocytosis (WBC 13.4 × 10^3^/μl) and mild anemia (Hb 10.8 g/dl). Computed tomography (CT) of the abdomen and pelvis revealed extraluminal gas near the rectal anastomosis, with gas tracking along the mesocolon into the retroperitoneum and mediastinum (Fig. 1A–C), findings consistent with iatrogenic perforation.

Given ongoing anticoagulation, immediate reversal was initiated with Idarucizumab.

**Figure 2:**
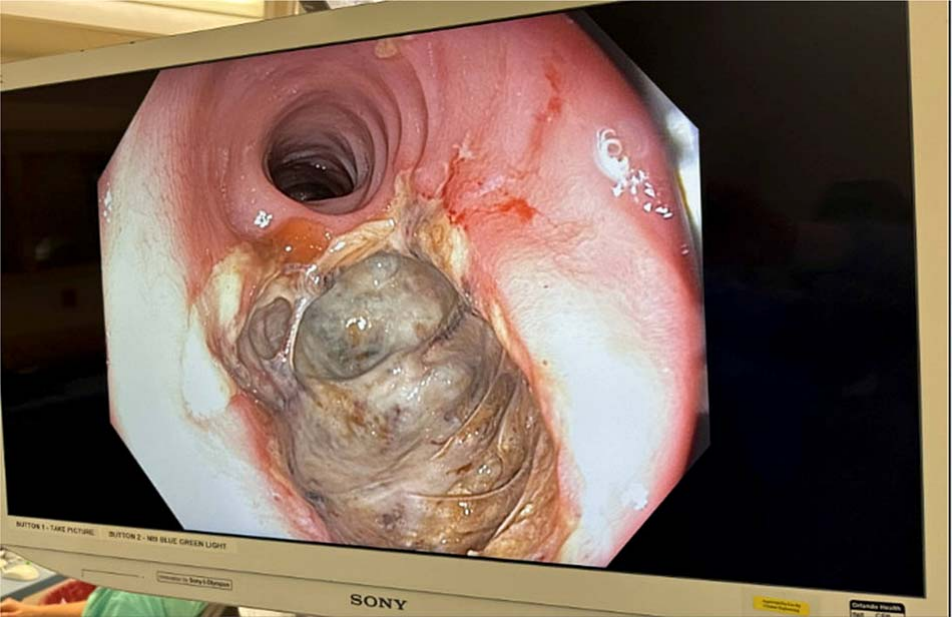
Initial findings on exam under anesthesia with flexible sigmoidoscopy revealing right lateral rectal wall disruption 8 cm from the anal verge, involving ~30% of the luminal circumference.

Examination under anesthesia with flexible sigmoidoscopy revealed a right lateral rectal wall disruption approximately 8 cm from the anal verge, involving close to 30% of the rectal circumference (Fig. 2). In the absence of peritonitis, the patient was admitted for observation and managed nonoperatively with bowel rest and intravenous ceftriaxone 1 g every 24 h, and metronidazole 500 mg every 12 h.

**Figure 3:**
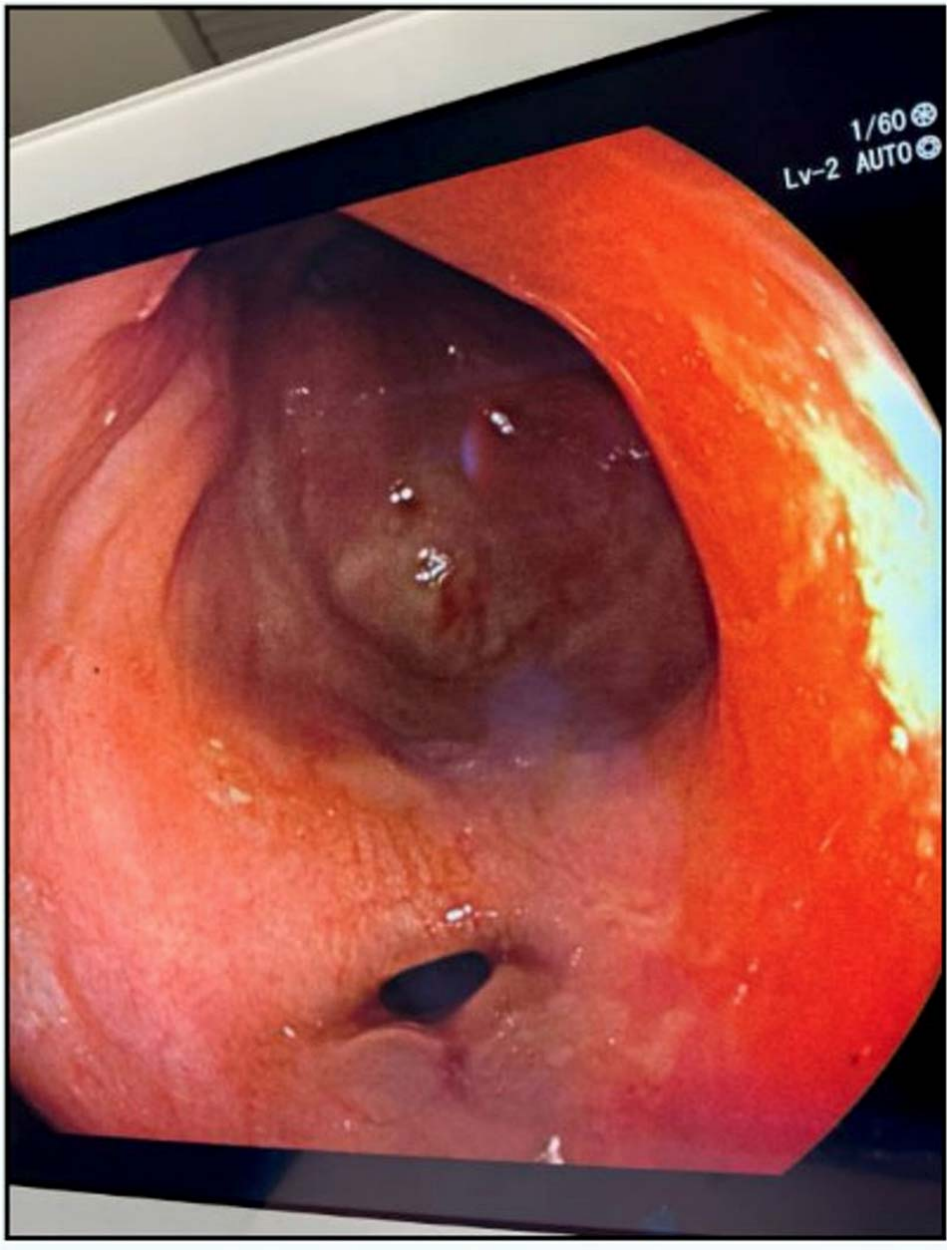
Follow-up flexible sigmoidoscopy at 4 weeks showing near-complete healing of the rectal perforation, with a small residual mucosal disruption.

**Figure 4:**
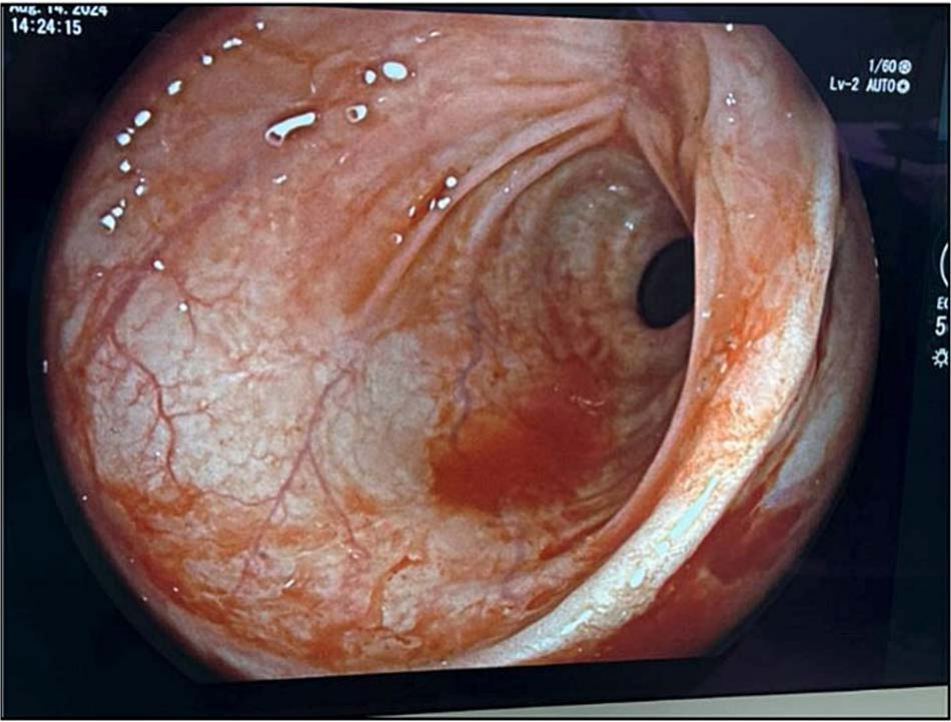
Flexible sigmoidoscopy at 6 weeks demonstrating complete mucosal healing and re-epithelialization at the prior perforation site, with no residual defect or inflammation.

The patient’s leukocytosis resolved during hospitalization, and she was discharged on hospital day 5 with dietary modifications, including a low-fiber diet. At 4-week follow up, flexible sigmoidoscopy demonstrated near-complete healing of the rectal perforation, with only minimal residual mucosal disruption (Fig. 3). By 6 weeks post-injury, follow-up flexible sigmoidoscopy confirmed complete mucosal healing and restoration of normal rectal wall architecture, though mild rectal stenosis persisted (Fig. 4). The patient remained asymptomatic with resolution of her initial symptoms.

## Discussion

Rectal perforation following ARM is a rare but clinically significant. This case illustrates how patient-specific factors can increase the risk of iatrogenic injury. Our patient had a complex surgical history, including multiple procedures for recurrent rectal prolapse, resection rectopexy, and perineal proctectomy with sphincteroplasty, which resulted in a stricture between two anastomoses. This segment is particularly vulnerable due to compromised blood supply at the junction of two hindgut anastomoses [6, 7] with increased susceptibility to mechanical trauma during the catheter insertion. Chronic anticoagulation added to the risk, as evidenced by immediate post-procedural bleeding.

Overdistension from balloon inflation is a rare mechanism of ARM-induced perforation [2]. Lee *et al*. recommends halting inflation if the patient does not perceive maximum pressure at 100 cc of saline or reports discomfort [2]. Zhao *et al*. suggest limiting inflation to 200 cc and improving operator training to minimize complications [4]. These considerations emphasize the need for cautious, patient-tailored ARM protocols, especially in high-risk individuals.

The management of anorectal perforation is guided by injury size and location, patient stability, and the presence of peritonitis. Extraperitoneal rectal injuries, such as in this case, can often be managed nonoperatively if the patient remains stable and without signs of sepsis or peritonitis [8]. The Eastern Association for the Surgery of Trauma conditionally recommends conservative management, including bowel rest, intravenous antibiotics, and close monitoring, with proximal diversion reserved for select patients with larger injuries or systemic instability [8]. Similarly, the World Society of Emergency Surgery emphasizes that in the absence of peritonitis or ongoing hemorrhage, conservative treatment can be effective, with surgical consultation and serial imaging to detect delayed complications [9].

Our patient’s use of dabigatran presented an added layer of complexity. Although bleeding was not brisk, reversal was performed out of caution due to perforation, her advanced age and multiple comorbidities. The American College of Gastroenterology advises individualized management of anticoagulation around endoscopic procedures, reversal agents are typically reserved for active bleeding or high-risk interventions [10].

Broad-spectrum IV antibiotics were provided to cover likely polymicrobial contamination, and bowel rest minimized the injured tissue from mechanical stress. Recent evidence from a multi-institutional study on traumatic rectal injuries suggests that routine presacral drainage and/or distal rectal washout offer no benefit and may increase complications, reinforcing a tailored, less invasive approach in carefully selected patients [11]. Notably, in the context of iatrogenic injuries from procedures such as ARM or colonoscopy, rather than traumatic high-energy injuries, the threshold for conservative management is even lower when the patient remains clinically stable [4, 9].

While ARM is generally considered a low-risk procedure this case demonstrates that iatrogenic complications can occur, particularly in elderly patients, with altered anatomy and additional risk factors such as chronic anticoagulation. Proper evaluation for patients with anorectal complaints should always begin with a physical exam, particularly a digital rectal exam, to identify structural abnormalities before proceeding to any form of instrumentation. Small, contained perforations can often be managed conservatively with close monitoring and early involvement of surgical teams, avoiding the morbidity associated with surgical intervention.

Ultimately, this case highlights the importance of thorough risk–benefit discussions before procedures such as ARM in high-risk patients, with particular attention to surgical history, tissue integrity, and anticoagulation status.
